# The risk of thyroid cancer in relation to residential proximity to nuclear power plants: a systematic review and meta-analysis

**DOI:** 10.1186/s12940-024-01143-6

**Published:** 2024-11-29

**Authors:** Susanna Abraham Cottagiri, Will King, Laura Rodriguez-Villamizar, Paul J. Villeneuve

**Affiliations:** 1 Department of Public Health Sciences, School of Medicine, Queens University, 99 University Ave, Kingston, ON K7L 3N6 Canada; 2 Faculty of Health, Industrial University of Santander, Cra. 32, Santander, Bucaramanga #29-31 Colombia; 3 Department of Neuroscience, Health Sciences Building, Carleton University, 1125 Colonel By Drive, Ottawa, ON K1S 5B6 Canada

**Keywords:** Thyroid cancer, Nuclear power plant, Ionizing radiation, Systematic review and meta-analysis

## Abstract

**Introduction:**

Ionizing radiation is a human carcinogen, and there is a public concern but limited evidence that it increases the incidence of cancer among those who live near nuclear power plants (NPPs). Previous analyses of thyroid cancer in these populations have been inconsistent, and the last synthesis was published nearly a decade ago. To address these gaps, we undertook a systematic review and meta-analysis.

**Methods:**

A search strategy was developed and applied to PubMed, Scopus, and Web of Science databases. A total of 2006 publications were identified, with 11 studies of thyroid cancer incidence that met the inclusion criteria. Study quality was assessed using the Office of Health Assessment and Translation (OHAT) tool. Summary risk estimates relating residential proximity to the NPPs and thyroid cancer were generated using a random effects model. Heterogeneity in the risk estimates was assessed for study features that included: distance to the NPP, study quality, and biological sex.

**Results:**

The 11 studies were categorized as either highly (*n* = 8) or plausibly (*n* = 3) prone to bias, primarily due to the reliance on ecological study designs. The meta-analysis summary relative risk of thyroid cancer among those who live close to NPPs (defined by ≤ 25 km distance or jurisdictional areas (e.g., community, county) relative to those who lived further away was 1.09 (95% CI: 0.93–1.29). The risk estimates were higher for studies that modelled more proximal residential distances (≤ 5 km) to NPPs than larger distances (≤ 25 km and jurisdictional areas). We found that the summary risk (RR=1.29, 95% CI: 0.77-2.16) was stronger among those studies less prone to bias. A non-significant increased risk was found among both men and women, but there was no evidence of sex differences in risk.

**Conclusion:**

Overall, the findings suggest that living near a nuclear power plant increases the risk of thyroid cancer. The small number of studies on this topic, and the finding of higher risks in studies less prone to bias highlights the need for better-designed studies.

**Supplementary Information:**

The online version contains supplementary material available at 10.1186/s12940-024-01143-6.

## Introduction

Over the last several decades, there has been a substantial increase in thyroid cancer worldwide, namely, the age-standardized incidence rate (ASIR) increased from 2.11 per 100,000 person-years in 1990 to 3.15 in 2017 [1]. This is predominately due to the increased capability of modern medical imaging being able to identify more cases of papillary carcinoma as well as increased surveillance [2, 3] and to a lesser extent due to other speculative factors such as increases in endocrine disrupting pollutants (eg: pesticides, phthalates compounds of flame retardants, and polyhalogenated aromatic hydrocarbons) [4] and increases in exposure to radiation from environmental (nuclear energy, industrial activity, etc.) [5] and medical sources [6, 7].

Increased exposure to ionizing radiation from a population-based perspective also occurs among individuals who live near nuclear power plants (NPPs). These plants release several gaseous and liquid radioactive effluents during routine operations [8]. Although NPPs tend to be located outside metropolitan areas, over time communities near these plants often experience increased population growth and urbanization due to employment, infrastructure, and urban sprawl [9]. It follows that a greater number of individuals are living near these plants, with the potential to be exposed to prolonged low doses of ionizing radiation, despite increased radiation protection measures implemented since the 1980s [10]. Even though exposure levels are low and not expected to exceed prescribed limits *(1 millisievert (mSv) per calendar year – effective dose)* [11], individuals living around NPPs are uneasy about the possible health risks, especially cancer, due to exposure to radiation [12, 13]. This public concern is due to positive findings from a series of epidemiological studies that attracted widespread media attention [14]. One of the most prominent studies was the health-district level ecological study that found an increased risk of childhood leukemia near a large nuclear fuel reprocessing site in Sellafield, UK in the 1980s [15]. Subsequent epidemiological studies of populations living near NPPs showed mixed results for childhood leukemia [16–23], and among adults, for other cancer sites such as thyroid [24, 25] and breast [24–27]. As a whole, these findings have not alleviated the concerns of residents [28, 29].

Individuals can be exposed to ionizing radiation either i) externally through high energy radiation (e.g., gamma radiation) that penetrates the human body or ii) internally from inhalation or ingestion of radionuclides (e.g., iodine-131,cesium-134, beryllium-7, potassium-40 etc.) that emit radiation [30]. While this exposure to ionizing radiation is low, the International Agency for Research on Cancer has classified this exposure as a human carcinogen [31] that increases the risk of developing several cancers including those of the thyroid [26, 32], breast [24, 25], bladder [33], lung [24], and kidney [26]. At this time, there is support for a linear no-threshold model implying that low levels of exposure may increase cancer risk [34].

Thyroid cancer is particularly relevant to ionizing radiation, as the thyroid gland is a highly radiosensitive organ [31, 35]. Radioisotopes of iodine are of significant concern for thyroid cancer. The primary biological mechanism underlying this sensitivity relates to the thyroid gland’s need for iodine from the bloodstream to produce hormones that regulate energy and metabolism. However, the gland is unable to distinguish between stable and radioactive iodine during this process [36]. A key iodine isotope of interest for thyroid cancer (although of only a physical half-life of 8 days) that is released from an NPP is Iodine-131 – this is a volatile radionuclide, that can be inhaled or ingested and can accumulate in the thyroid [37].

Residents who live near NPPs are exposed to ionizing radiation primarily from discharged radionuclides (internal exposure) such as elemental tritium (HT), tritium oxide (HTO), carbon-14 (C-14), iodine-131 etc., [38, 39] and the effective doses are estimated to be in the range of 0.0004 mSv/year [38] to 0.052 mSv/year [39]. Comparatively, these doses are much lower than dose estimates from higher-exposure populations such as the International Nuclear Workers Study (INWORKS) (17.4 mSv mean cumulative colon and lung dose) [40] and Japanese atomic bomb survivors (mean dose ~ 200 mSv) [41].

Epidemiologic studies support an excess risk of cancer in relation to prolonged exposure to low-dose ionizing radiation [42]. However, assessing carcinogenicity for prolonged low-dose exposure to ionizing radiation is extremely challenging in observational studies as it requires long follow-up periods to account for etiologically relevant exposure windows and to identify sufficient cancer cases [8, 43]. Consequently, studies of thyroid cancer risk among populations living near NPPs have mostly been ecological in nature with no individual-level data. Findings from these studies have been inconsistent with some studies reporting increased risks [26, 32, 44] while others not [45, 46]. There are several possible explanations for the heterogeneity in the risk estimates across studies, including exposure characterization, study quality (heterogeneity in methodology, analysis etc.), and biological sex differences in susceptibility.

Sex differences in thyroid cancer risk have been reported in populations living near NPPs, with some studies showing higher thyroid cancer risks in women than men [25, 47] whereas other studies have shown the opposite [33]. The biological mechanisms that could explain these differences are not established, but it has been suggested that sex differences in susceptibility are due to the role of gene variation in DNA damage/ repair [48], polymorphism in estrogen receptors [49], sex-chromosomal features [50], and hormonal regulation [48]. Women have higher background thyroid cancer rates suggestive that they are more susceptible than men [51]. Perhaps most compelling is the evidence of a higher thyroid cancer risk per same unit dose increase in women relative to men [52]. Understanding sex differences in risk is important for not only strengthening conclusions for characterizing risks for subgroups, but also for providing insights into underlying biological mechanisms that contribute to differential susceptibility.

Findings from past studies of thyroid cancer in relation to residential proximity to NPP have undoubtedly been influenced by exposure measurement error due to methodological challenges (low population and cases) and the reliance on ecological designs. Past studies tended to classify exposed populations as those living within relatively large buffers from NPPs (e.g., ≤ 20 km [33] or ≤ 25 km [39]), or jurisdictional areas (e.g.: community, county, municipality etc.) [26, 44, 46]. This spatial resolution may be inadequate as highlighted by findings from exposure studies that used advanced air dispersion models that incorporate meteorological parameters such as wind speed and direction that have shown radiation exposures are substantially higher for residences within 5 km of an NPP [38, 39]. It follows that there is an important need to evaluate the heterogeneity in risk estimates by residential distance to the NPP to best identify those at risk.

To date, there has been one systematic review of this topic [53]. This study by Kim et al. considered publications on residential proximity to NPPs and thyroid cancer up to March 2015, and conducted a meta-analysis on 13 studies (10 incidence and 4 mortality). Overall, this review found no increased risk with a standardized incidence ratio (SIR) of 0.98 (95% CI: 0.87 – 1.11), however, a statistically significant increased risk was observed among studies that restricted to risk estimates derived among populations living within 20 km of an NPP (OR = 1.75; 95% CI: 1.17 – 2.64). Additionally, this review reported no increased risks for subgroup analyses, specifically considering biological sex and types of reference populations [53].

The previous meta-analysis was published in 2016 and several papers with updated follow-up periods, or detailed breakdown of risk estimates have since been published [33, 47, 54]. This paper sought to provide an updated systematic review and meta-analysis focusing on thyroid cancer incidence while assessing sources of heterogeneity, specifically by subgroups of exposure definition, biological sex, and study quality.

### Methodology

This review follows the Preferred Reporting Items for Systematic Reviews and Meta-Analyses (PRISMA) guidelines [55]. The protocol of this review was registered on the International Prospective Register of Systematic Reviews (PROSPERO) in October 2022 (registration number: CRD42022364057) [56].

### Eligibility criteria

To formally identify the exclusion and inclusion criteria, a search strategy was formulated using the Population, Exposure, Comparator, and Outcomes (PECO) framework [57]. *Population:* Humans of all ages and sexes were included. *Exposure:* The exposure of interest was residential proximity to NPPs and this was defined according to several measures including: distance buffers, residency in administrative units (county, town, municipalities etc.), or exposure estimations (such as doses estimates from air dispersion models) for individuals living near these plants during routine operations. *Comparator:* Three types of comparisons were of interest including i) comparisons based on proximity to the NPP (usually defined by distance buffers) ii) comparisons based on jurisdictional areas near a NPP and further away defined by areas, towns, municipalities, counties, etc., and iii) comparisons based on dispersion model exposures. *Outcomes:* Thyroid cancer incidence among all ages was the outcome of interest. However, all cancers among all ages were considered relevant during the search process, as some studies report all cancers in the title and abstract but provide risk estimates by cancer site in the manuscript. Thyroid cancer mortality studies were excluded because unlike cancer sites with poor prognosis such as lung cancer where incidence can estimate mortality [58], 80—85% of all thyroid cancer cases are papillary thyroid cases [59], which are slow-growing cancers with a high survival rate [60]. Consequently, mortality studies of cancers with high survival rates such as thyroid cancer may be prone to bias.

All article types other than reviews, including letters, commentaries, and short articles were considered eligible if they contained relevant estimates of relative risk. Lastly, we included only studies published in English.

### Search strategy and databases

The search strategy for three indexed databases PubMed, Scopus, and Web of Science was developed in consultation with a health librarian (H.M.) at Carleton University. A shortlist of 10 articles that met the eligibility criteria was tested for validity using the search strategy that was developed. Databases and corresponding search terms used for the final search strategy—are provided in Supplementary Table 1. The search strategy was tested on PubMed using Medical Subject Headings (MeSH) and free-text terms and then replicated on two other databases (i.e., Scopus, and Web of Sciences (WOS)). Lastly, we included papers published from 1983 till November 2nd, 2023.

### Screening

**Fig. 1:**
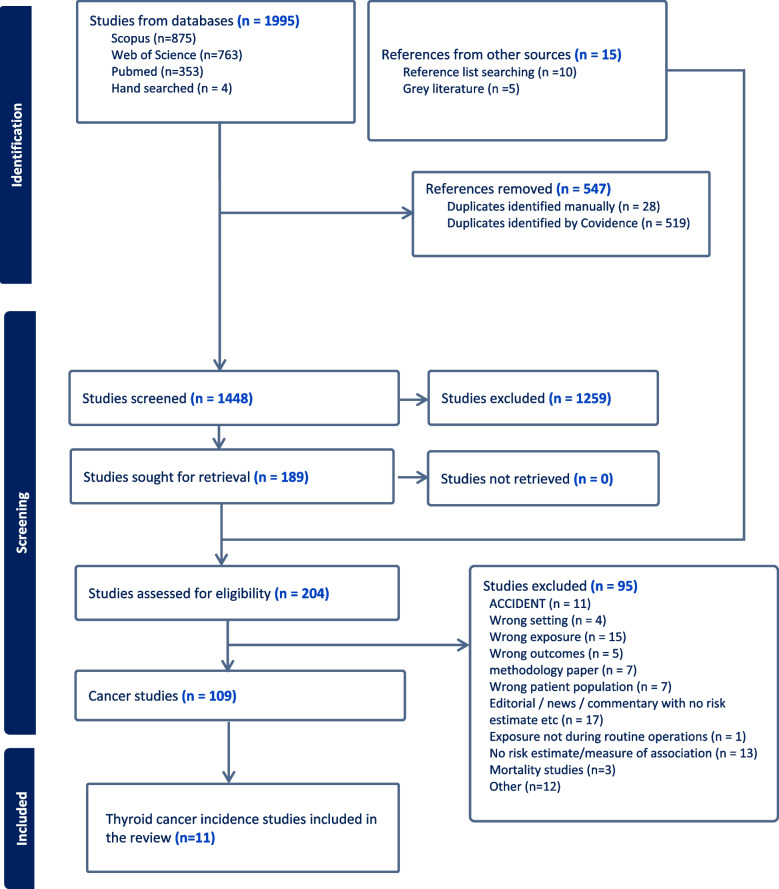
PRISMA flowchart depicting study selection process

Studies identified from these databases were imported into Covidence software [61]. Duplicates were removed by Covidence. Two reviewers (S.A.C. and G.G.) screened the titles and abstracts, and conflicts were resolved by an expert in environmental epidemiology (P.V.). Subsequently, identified articles went through full-text screening and conflict resolution by the same reviewers. Additionally, a reviewer (S.A.C.) screened the reference list of retained articles following full-text review to identify any possible additional missing articles. Figure 1 presents the PRISMA diagram detailing the screening process, and the number of articles at each stage of the review.

### Data extraction

A tailor-made data extraction template was created using Covidence. For each article, a reviewer (S.A.C.) extracted relevant data while the accuracy and missingness of these data were checked by one of two reviewers (G.G. or C.W.). The data extracted included: 1) article title, 2) lead author, 3) year published, 4) country/region of the study population, 5) study design, 6) type of nuclear facility and number of facilities, 7) study period, 8) type of exposure measurement (distance, dose estimate etc.), 9) exposure measurement and comparison 10) study population 11) type of regression model and covariate adjustment and finally 12) risk measures and endpoints by cancer site, male, female and age were extracted separately when reported.

For cohort and ecological studies, we extracted RRs, HRs, or SIRs as effect estimates. There were several instances where we combined risk estimates within a single study to obtain an overall summary risk estimate. For example, when risk estimates were provided only for i) nested buffers (non-overlapping and concentric zones eg: ≤ 5 km, > 5 – 10 km, etc.) ii) individual nuclear sites and iii) sex-specific. For all the above instances, data across categories were collapsed using a random effects model (DerSimonian and Laird) [62]. Three studies had female only estimates. Two studies [46, 63] did not provide risk estimates for males and for one study [64] we were unable to combine risk estimates across 5 km equidistant buffers for males to obtain an overall risk. This is because there were zero cases and subsequently no confidence intervals for four of the five buffers for the male sub-group.

When data/populations used in multiple publications overlapped, we selected the study with the longest follow-up period, most cases, or those that provided more detailed risk estimates (e.g., male/female estimate separately rather than overall). Key characteristics of the included studies are given in Table 1.

**Table 1:** Characteristics of included studies in the meta-analysis for thyroid cancer incidence risk among populations residing in proximity to nuclear power plants

Lead author (year)	Study design	Country/ region	Number of facilities	Study period	Exposed population	Unexposed (reference) population	Age group	Sample size (cases)	Effect measure	Exposure measurement	Male/ Female	Relative Risk and 95% CI
Bazyka (2012) [26]	Ecological	Ukraine	3 NPPs	2003—2008	Observed Communities near NPPs Pivdennoukrainsk, Energodar, Netishyn	Ukraine	n.s	129,845 (49)	SIR	Area (community) comparison	Total	1.23 (0.89—1.58)
Boice (2009) [44]	Ecological	USA	1 NPP	1990—2004	Two counties Armstrong and Westmoreland in Western Pennsylvania	Six comparison counties (Clarion, Clearfield, Somerset, Beaver, Washington, Erie)	n.s	(433)	RR	Area (county) comparison	Total	0.88 (0.78—0.99)
								(102)			Male	0.86 (0.68—1.09)
								(331)			Female	0.88 (0.77—1.01)
Bunch (2014) [65]	Birth cohort	United Kingdom	1 NPPs	1950 and 2006; 1963—2006	Cumbrian births between 1950 and 2006	National incidence for 1971—2006 using person-years analysis	0—57	338,119 (89)	SIR	Seascale ward as at 1981 + Allerdale and Copeland county districts excluding Seascale + Remainder of Cumbria	Total^a^	0.82 (0.45 – 1.50)
Demoury (2020) [54]	Ecological	Belgium	2 NPPs (Doel and Tihage)	2000—2014	Observed around buffer	Walloon Region for Tihange and Flemish population for Doel	All ages	n.s (33)	RR	0—5 km^a^	Total	0.81 (0.69 – 0.95)
								n.s (68)		0—20 km^a^		0.82 (0.78 – 0.86)
Desbiolles (2017) [33]	Ecological	France	7 NPPs	1995—2011	Exposed to 1 NPP site	Municipalities with no NPP or other nuclear installations within 20 km of their town hall	> 15	445,935 (671)	RR	0 – 20 km	Total^a^	0.97 (0.75 – 1.26)
								(186)			Male	1.12 (0.95—1.32)
								(485)			Female	0.86 (0.77—0.96)
Gulis (1998) [64]	Ecological	Slovakia (Trnava)	1 NPP	1986–1995	District of Trnava	European population	n.s	3456 (1)	SIR	≤ 5 km	Female	1.51 (0.00—3.03)
								112,092 (22)		0 – 20 km^a^		1.05 (0.83 – 1.34)
								75,618 (5)		10 – 15 km	Male	1.64 (0.89 – 2.39)
Kim (2018) [47]	Cohort	South Korea	4 NPPs	1992—2006	Residing within 5 km and 0 – 30 km of an NPPs	Residing > 30 km of an NPP	> 20	36,176 (54)	HR	≤ 5 km	Total	3.20 (1.73—5.93)
								n.s (166)		0—30 km^a^		2.91 (1.86- 4.53)
								(10)		≤ 5 km	Male	3.38 (0.92—12.39)
								(35)		0—30 km^a^		3.73 (1.49 – 9.28)
								(44)		≤ 5 km	Female	3.15 (1.56—6.34)
								(131)		0—30 km^a^		2.69 (1.62 – 4.47)
Lane (2013) [39]	Ecological	Canada	3 NPPs	1990—2008	Those who lived within 25 km of 3 NPPs in Ontario	Ontario general population	All ages	1,984,500 (4591)	SIR	0 – 25 km	Total^a^	1.11 (0.87 – 1.43)
											Male^a^	1.41 (1.32—1.51)
											Female^a^	1.06 (0.79—1.42)
Salerno (2016) [63]	Ecological	Italy	1 NPP	2002—2010	Observed in Trino municipality where former NPP was located	Turin City	All ages	ns	SIR	Area (community) comparison	Female	1.86 (1.06—2.66)
Wang (2016) [66]	Ecological	Taiwan	3 NPPs	1979—2003	Plant-vicinity (< = 14.2)	Non-plant-vicinity (> 22.8)	n.s	ns	RR	≤ 14.2 km	Total	0.79 (0.12 -5.28)
Zadnik (2008) [46]	Ecological	Slovenia	1 NPP and 1 nuclear waste repository site	1984–2003 (Krško NPP started operations in 1984)	Spodnjeposavska statistical region	Slovenia	n.s	ns	SIR	Area (region) comparison	Female	0.94 (0.67 – 1.28)

^a^Combined weighted summary estimate

*n.s* not specified

### Study quality

To assess the quality of the identified studies, we used the Office of Health Assessment and Translation (OHAT) tool developed by the National Toxicology Program, National Institute of Environmental Health Sciences (NIEHS) [67]. This risk of bias tool is widely used for assessing the quality of environmental health studies [68–70]. Briefly, the tool categorizes biases (domains) from each study into one of four risk of bias (ROB) groups - definitely low ROB, probably low ROB, probably high ROB, definitely high ROB. Each study is then classified into one of three tiers: tier 1 indicates low risk of bias, tier 2 plausible risk, and tier 3 high risk of bias. We considered exposure characterization and confounding bias as the key domains whereas selection bias, attrition/exclusion bias, outcome assessment, selective reporting, and appropriate statistical methods were considered as the other domains. Tier 1 studies were those where all key and most other domains scored definitely low ROB or probably low ROB. Tier 3 studies were those where all key domains and some other domains scored probably high ROB, or definitely high ROB. Tier 2 studies are those that fell into neither tiers 1 or 2 [67]. Two reviewers (S.A.C. and L.R.) scored the papers independently and deliberated to resolve conflicts and reach a consensus risk of bias score where there was disagreement. Table 2 provides results of the bias analysis and Supplementary Table 2 provides the criteria that were used for scoring the observational studies.

**Table 2:** Assessment of bias using the Office of Health Assessment and Translation (OHAT) tool

Author (Year)	Selection bias	Confounding bias	Attrition/ exclusion bias	Exposure characterization	Outcome assessment	Selective reporting bias	Appropriate Statistical Methods	Overall Study Confidence
Bazyka (2012) [26]	+ +	–	+ +	–	+ +	+ +	-	Tier 3
Boice (2009) [44]	+	-	+ +	–	+	+ +	-	Tier 3
Bunch (2014) [65]	-	–	-	–	-	+ +	-	Tier 3
Demoury (2020) [54]	+ +	-	+ +	-	+ +	+ +	+	Tier 2
Desbiolles (2017) [33]	-	-	+ +	–	+ +	+ +	+ +	Tier 3
Gulis (1998) [64]	+ +	–	+ +	-	+ +	+ +	-	Tier 3
Kim (2018) [47]	+ +	+ +	+ +	-	+ +	+ +	+ +	Tier 2
Lane (2013) [39]	+ +	-	+ +	+	+ +	+ +	-	Tier 2
Salerno (2016) [63]	+	–	+ +	–	+	+ +	-	Tier 3
Wang (2016) [66]	+ +	–	+ +	-	+ +	+ +	-	Tier 3
Zadnik (2008) [46]	+ +	–	+ +	–	+ +	+ +	-	Tier 3

+ +Definitely low risk of Bias

+Probably low risk of Bias

-Probably high risk of bias

–Definitely high risk ofbias

### Statistical analysis

An overall summary risk estimate and the corresponding 95% confidence interval along with a forest plot were generated using the random-effects model (DerSimonian and Laird) [71]. A random effects model was chosen as the studies varied on several aspects including exposure characterization, outcome measurement, population characteristics, and adjustment for confounders [72]. For the overall summary effect estimate, we used the most commonly reported distance buffer across studies, this was either jurisdictional area level comparisons or large buffers such as ≤ 25 km, ≤ 30 km etc. The I-square statistic and its corresponding *p-*values were used to characterize the heterogeneity in the measures of association across the studies [71]. Egger’s test and funnel plot were used to assess potential publication bias.

Next, we categorized studies into three groups based on the most proximal exposure characterization (smallest reported buffer) reported in each study. The three groups were i) studies that defined the exposed population as within 5 km of an NPP, ii) studies that used residential buffers within 25 km of an NPP, and iii) those that relied on jurisdictional measures of proximity (e.g., town, county, etc.). No studies using dispersion models of exposure were identified. For each of these three exposure metrics, the weighted summary risk estimates and the corresponding 95% confidence interval along with a forest plot were generated using the random effects model. Based on these results, the smallest buffer reported was used to assess heterogeneity among subgroups of biological sex and study quality in an attempt to capture the most etiologically relevant risk estimates reported in each study. For all the subgroup analyses, the Cochran’s Q statistic [73] was used to assess heterogeneity in the summary measure of association between the groups. This test provides a probability based on the chi-square distribution indicating the likelihood of variation across studies within each subgroup [73].

All analyses were performed using Stata version 18 (StataCorp LLC, College Station, TX, USA).

## Results

This systematic review identified 2,006 research articles, of which 24 reported risk estimates between residential proximity to an NPP and thyroid cancer (incidence or mortality) After removing overlapping study populations and mortality studies, 11 thyroid cancer incidence papers were retained for analysis (Fig. 1). Of the included papers, nine were ecological studies, one was a cohort study, and one a birth cohort study. The studies represented 10 countries (Belgium, Canada, France, Italy, South Korea, Slovenia, Taiwan, UK, Ukraine, and USA), 27 nuclear power plants, and 1 nuclear waste repository site (Table 1).

All papers were categorized as either highly (*n* = 8) or plausibly (*n* = 3) prone to biases, primarily due to exposure characterization or confounding biases. Exposure characterization varied among the studies as four used jurisdictional area comparisons (community, county, municipality, and region) [26, 44, 46, 63] while the others used variable buffers from ≤ 5 km [54, 64], ≤ 14.2 km [66], ≤ 20 km [33], ≤ 25 km [39]. The cohort study collected the length of residence (exposure duration) for living near an NPP (using 5 km, 5 – 30 km etc.) prospectively [47]. The birth cohort study retrospectively collected residential mobility related information for the identified cohort members in the counties of interest [65]. With respect to confounding, only one cohort study [47] could control for individual-level confounders (apart from age and sex). The OHAT risk of bias assessment summaries are shown in Table 2.

**Fig. 2:**
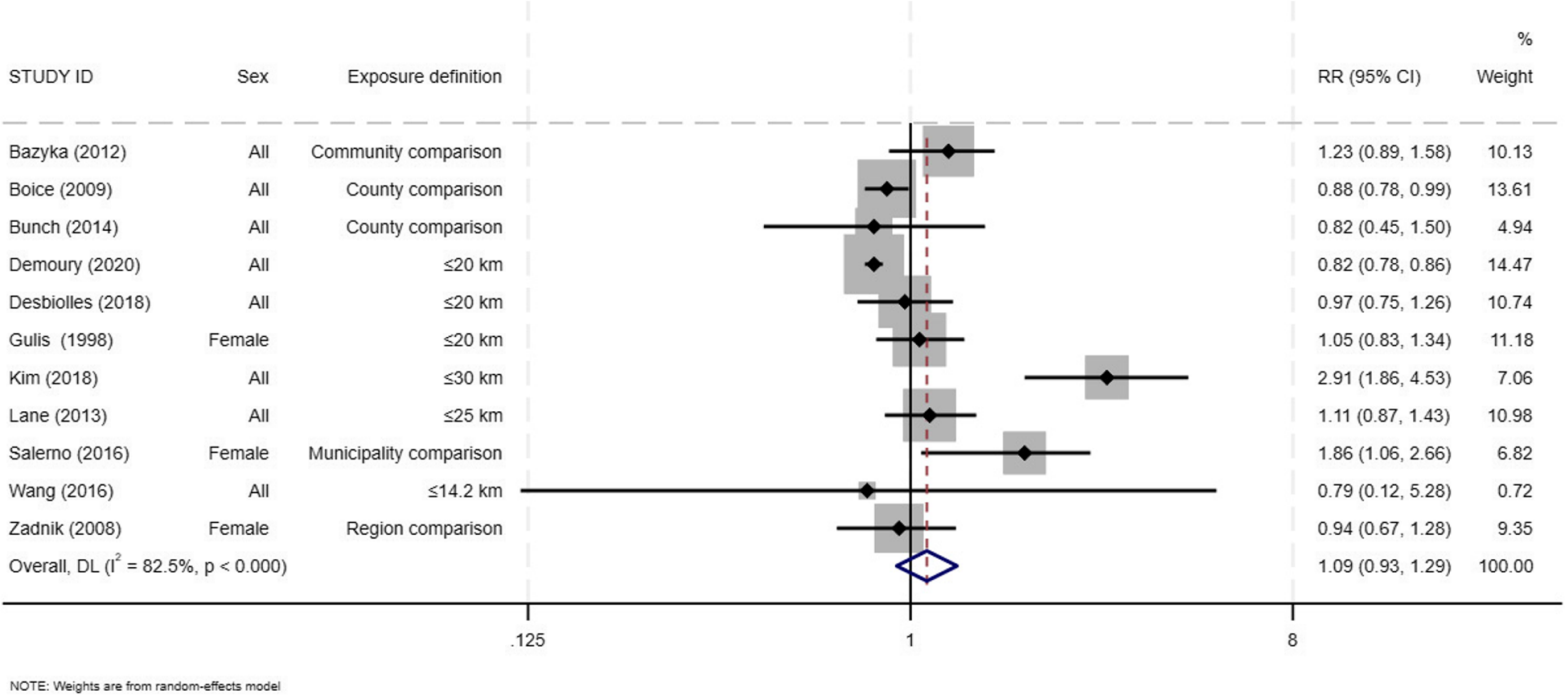
Forest plot for total incident thyroid cancer among residents in proximity to a nuclear power plants

The overall weighted summary estimate effect for the 11 incident thyroid cancer studies for those who live near an NPP was RR = 1.09 (95% CI: 0.93 -1.29) (Fig. 2). This measure of association across these studies exhibited a high degree of heterogeneity (I-square = 82.5%).

**Fig. 3:**
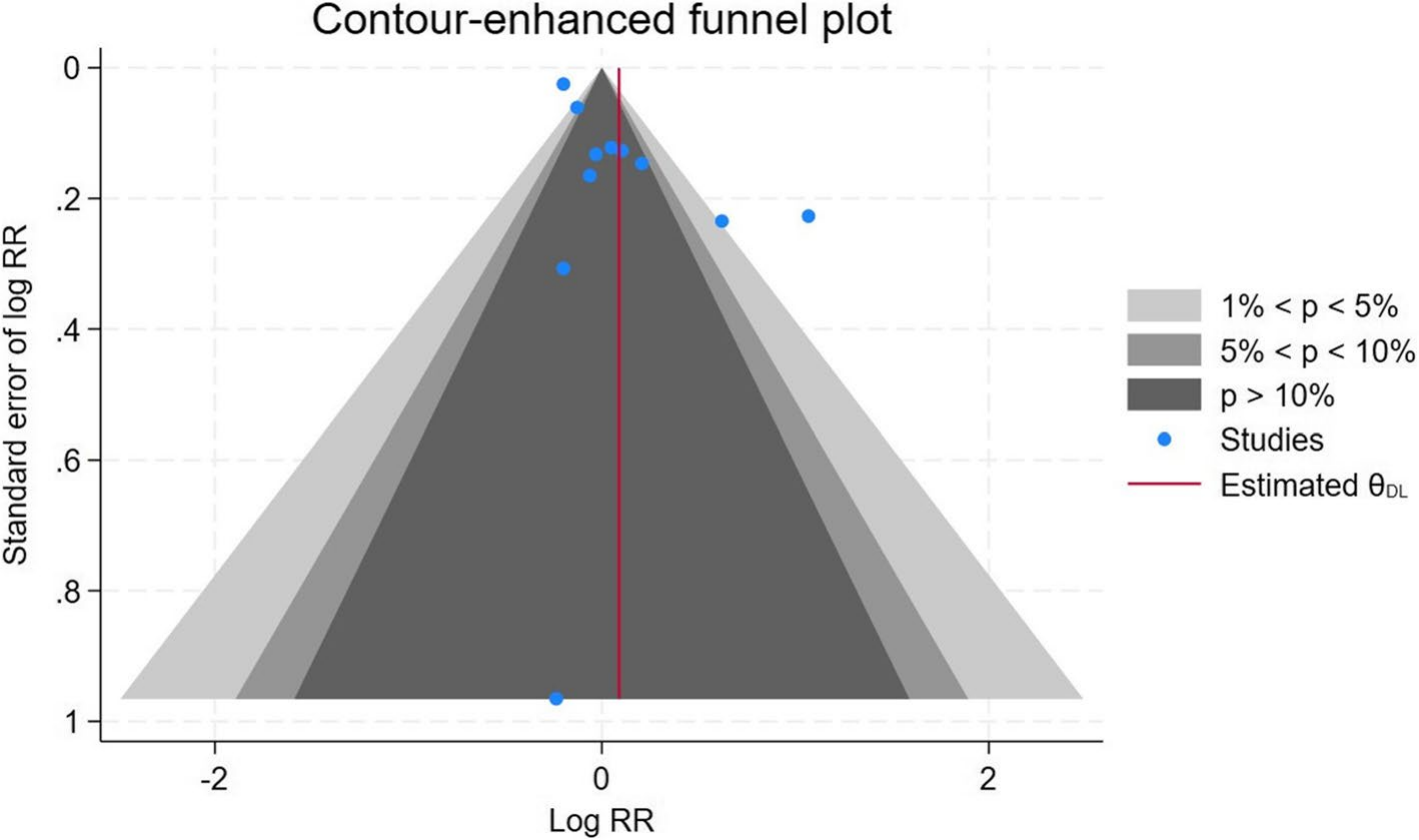
Funnel plot of incident thyroid cancer studies among residents in proximity to a nuclear power plant

The Egger’s test suggested the presence of publication bias (*p-*value =0.04), however, the trim and fill imputed no new studies - the corresponding funnel plot is shown in Fig. 3. We also conducted an analysis of influence, which showed that no single study exerted an undue influence on the summary measure (Supplementary Figure 1).

**Fig. 4:**
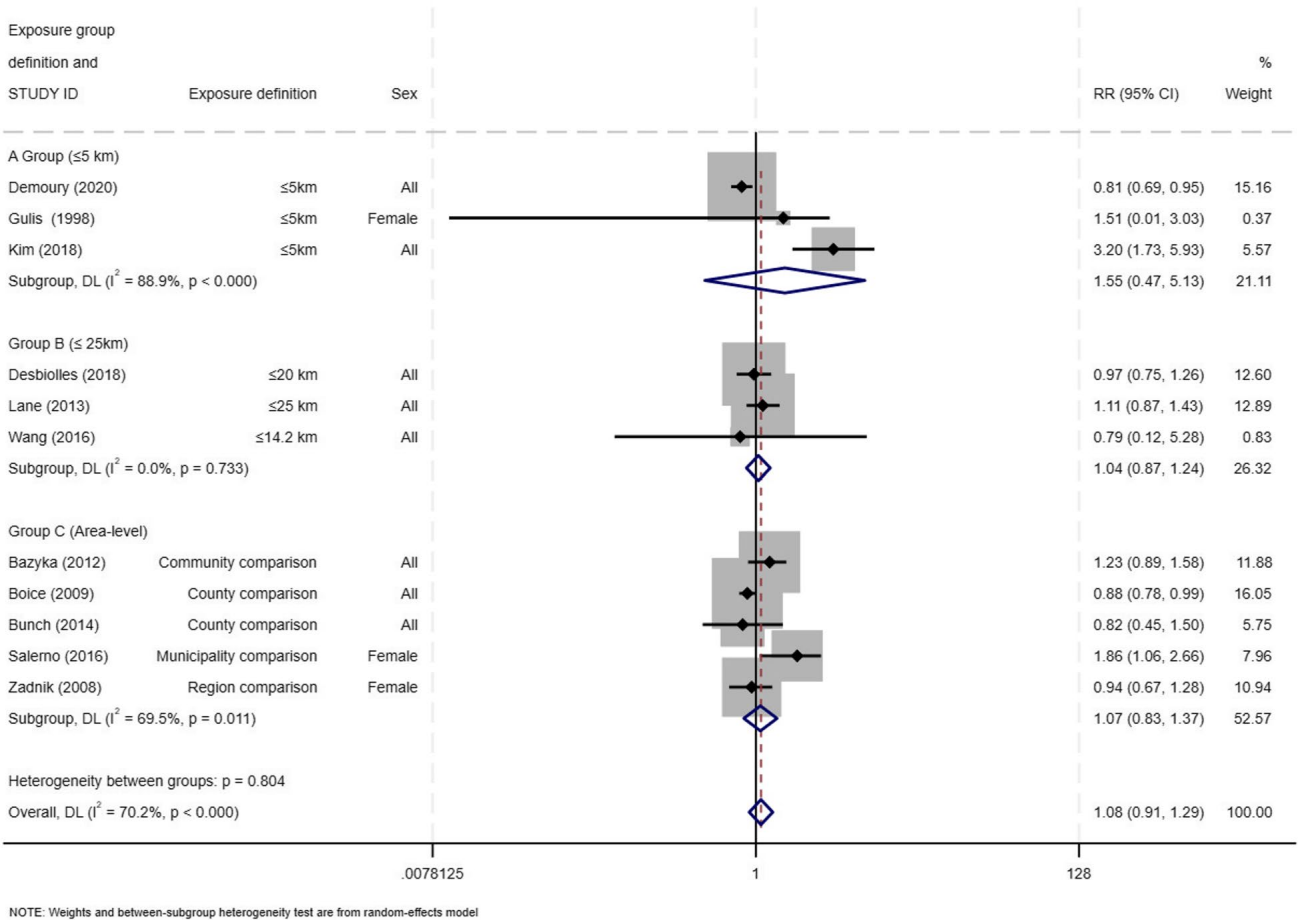
Forest plot for incident thyroid cancer among residents in proximity to a nuclear power plant using the most precise buffer reported

In the analysis of studies grouped by the smallest buffer reported in each study, we found that the sub-group of studies (*n* = 3) that were based on ≤ 5 km proximity to the NPP reported a stronger effect (RR = 1.55; 95% CI: 0.47 -5.13), when compared to findings derived with a buffer distance of ≤ 25 km (*n* = 3) (RR = 1.04; 95% CI: 0.87 -1.24) or by modeling jurisdictional areas (*n* = 5) (RR = 1.07; 95%CI: 0.83 -1.37) (Fig. 4). Even though these risk estimates were notably different across these three exposure definitions, the confidence intervals overlapped and Cochran’s Q statistic for heterogeneity between the subgroups was not statistically significant (*p-*value = 0.80; I-square = 70.2%).

**Fig. 5:**
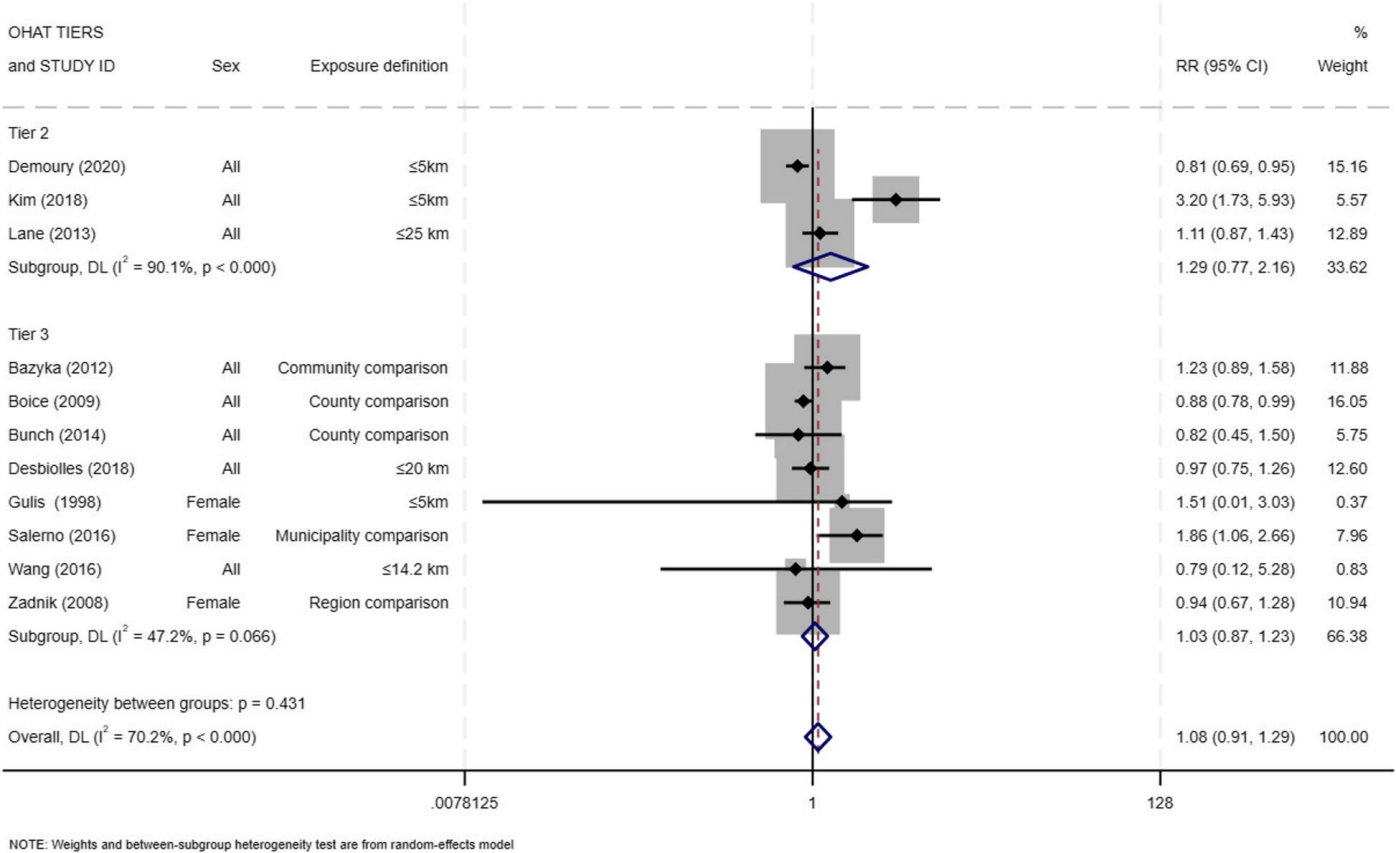
Forest plot for incident thyroid cancer among residents in proximity to a nuclear power plants by quality of studies

Next in the heterogeneity analysis by study quality, we found a more pronounced effect for studies plausibly prone to bias compared to studies highly prone to bias, however, the difference in these summary risk estimates was not statistically significant. The weighted summary estimate for studies that were plausibly prone to bias (Tier 2) was RR = 1.29 (95% CI = 0.77—2.16) and for studies highly prone to bias (Tier 3) was RR = 1.03 (95% CI = 0.87 – 1.23) (Fig. 5). The Cochran’s Q statistic for heterogeneity between the subgroups was not statistically significant (*p-*value = 0.43; I-square = 70.2%). There were no Tier 1 studies based on our OHAT assessment.

**Fig. 6:**
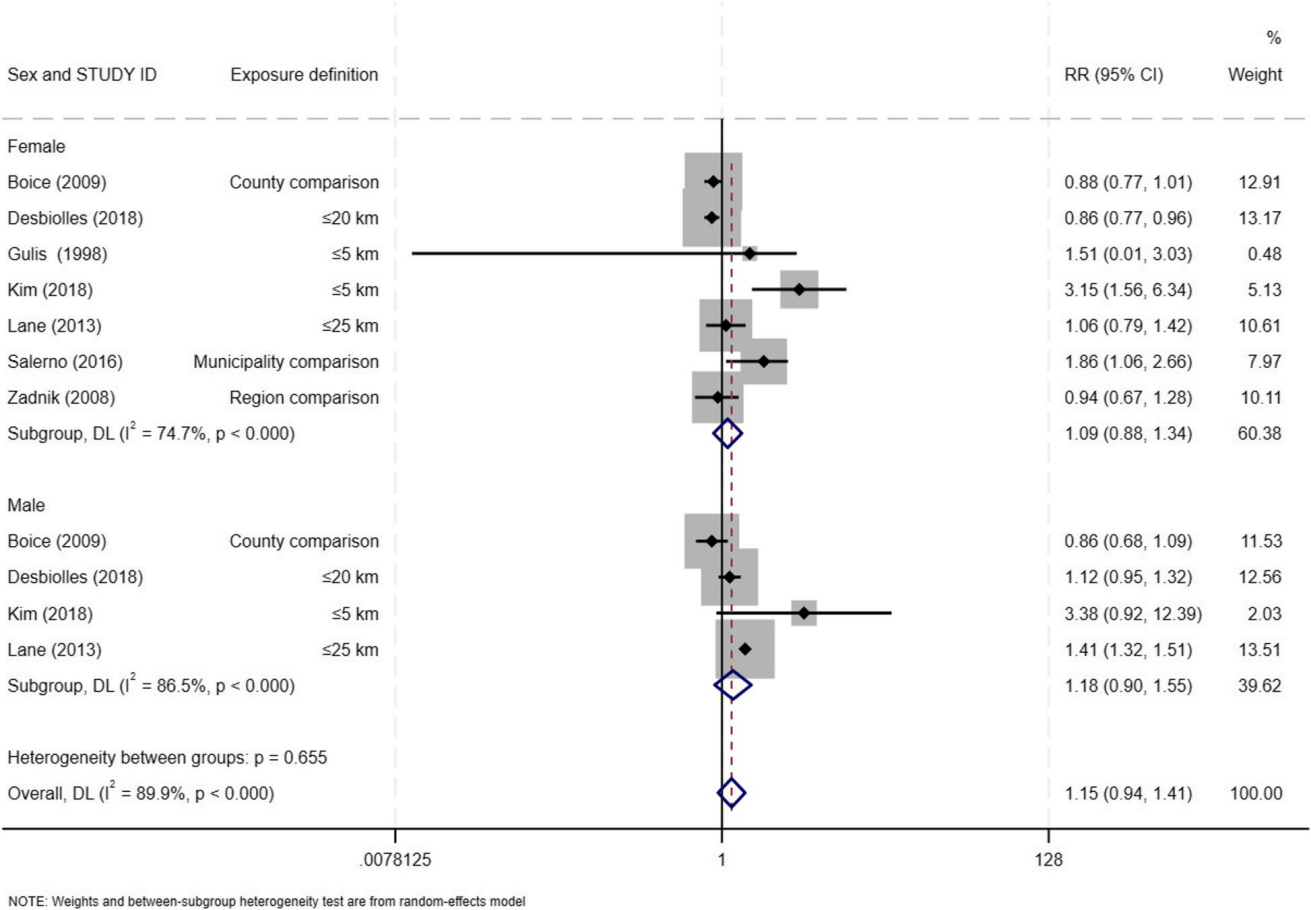
Forest plot for incident thyroid cancer among residents in proximity to a nuclear power plants by biological sex

Lastly, we found a non-statistically significant increase in risk for men and women, but no statistically significant difference in risk estimates was observed between the sexes (*p-*value = 0.66 and I-square = 89.9%). The weighted summary risk for men was RR = 1.18 (95% CI: 0.90 – 1.55) and for women was RR = 1.09 (95% CI: 0.88 – 1.34) (Fig. 6).

## Discussion

In this meta-analysis of 11 studies of incident outcomes, we found a non-statistically significant elevated risk of thyroid cancer risk for those who live near NPPs when compared to those who do not. The summary risk was stronger for the sub-group of studies whose risk estimates were calculated using smaller distance buffers. We also found that the summary risk estimates were stronger when restricted to studies less prone to bias. Lastly, we found no risk difference between men and women.

Our findings of a slightly increased risk of thyroid cancer for those who live near NPPs (RR = 1.09; 95% CI: 0.93 -1.29) differ somewhat from the previous meta-analysis where the summary risk estimate was essentially null (SIR = 0.98; 95% CI: 0.87 – 1.11) [53]. The difference in our summary estimates is due to the inclusion of three additional studies. This includes two studies published after the previous systematic review [63, 66] as well as one other study that was published before but excluded from their review [39]. The identification of these additional studies is likely due to our decision to apply our search across three databases, whereas the Kim et al. (2016) study [53] relied on Embase and Medline. Additionally, the present meta-analysis includes risk estimates derived from updated three cohorts that resulted in a higher number of incidence cancers [33, 47, 54].

The meta-analysis found substantial heterogeneity (I-square value > 80%) across the study-specific measures of association. This pattern can be explained by many reasons, including variability in the characterization of exposure, study quality (variability in methodology, type of models, adjustment factors in models), as well as differences in characteristics of the study population (e.g., age and sex distribution). Below, we discuss the results of heterogeneity analysis for three factors.

Firstly, our subgroup analyses suggest that some of the observed heterogeneity is due to study differences in exposure characterization by distance. Specifically, we observed an attenuation of risk estimates with increasing residential distances to the NPP. The risk estimate based on smaller distance buffers (≤ 5 km) were higher, however, the confidence interval for this summary measure was quite wide owing to the small number of such studies and the small number of identified cancers in these studies. These findings of a stronger risk estimate with shorter distances are in line with exposure modelling work which reports that radiation exposures drop off quickly with increasing distances to the NPP [74–76]. For instance, a recent French study reported distances to be inversely correlated to estimated dose for concentric circles of 5 km zones. Although within these equidistant zones, there was considerable variability due to meteorological and topographical factors, the effective dose for ≤ 5 km was 1.2 mSv per year compared to 0.04 mSv per year for the 15–20 km [75]. Additionally, in line with our findings, a review [77] that looked at a rare cancer (childhood leukemia) for those living near NPPs reported that the risk of living within a 25 km buffer was RR = 1.00 (95%CI: 0.95 -1.05) but the risk within 5 km was RR = 1.45 (95%CI: 0.74 -2.86) for case–control studies and RR = 1.33 (95%CI: 1.05 -1.68) for ecological/cohort [77].

Secondly, we hypothesized that some heterogeneity is due to differences in study quality (apart from spatial resolution in characterizing exposure). We found stronger risk estimates for studies less prone to biases compared to those highly prone to biases. Two key biases that impacted the quality of studies in our meta-analysis were non-differential misclassification of the exposure and confounding bias. Non-differential misclassification of the exposure may have affected most studies in our review. Summary estimates can be susceptible to yielding false positive estimates if poor quality studies are biased toward overestimating or false negative estimates if poor quality studies are biased toward underestimating [78]. In our meta-analysis, studies that were highly prone to bias were mostly ecological and, there was considerable misclassification of exposure (larger exposure assessment units). In these studies, individuals with lower exposure were grouped with higher exposure individuals, which likely biased most estimates to dilution and led to an underestimation of the adverse effect. This also correlates with the stronger effect we found in studies plausibly prone to biases and the diluted effect in those highly prone to biases. Our findings by study quality were similar to the previous meta-analysis that found a statistically significant association among higher quality studies that classified exposure as living less than 20 km from an NPP [53]. In relation to confounding bias, most studies in our analysis used aggregate levels of data, which in extension meant little to no ability to adjust for variables that could influence the association between thyroid cancer and living near a nuclear plant. Although this likely led to some residual confounding and biased estimates, it is important to note that there is little evidence for any risk factors that could confound the association between living near a nuclear power plant and thyroid cancer. Other than radiation [79], thyroid cancer related risk factors in the literature that could be relevant include being female [49], family history [80], higher body mass index [81], and iodine in the diet [82], however, evidence for these factors are contradictory [79]. 

Lastly, apriori we hypothesized that some heterogeneity in the risk estimates is due to differences in the sex distribution across study populations. Studies of populations with variable exposure levels have reported differential susceptibility by biological sex in the development of thyroid cancer when exposed to ionizing radiation [83]. However, our meta-analysis found no statistically significant difference in the summary measure of effects between the two groups. Our findings are likely due to three factors i) the ecological nature of most studies which extends to the inability to assign exposures at the right etiological window, ii) the lack of individual-level data, and iii) uncontrolled confounding for thyroid cancer related risk factors, although supported by mixed evidence for factors such as diet [82], family medical history [79, 80], and lifestyle factors [79]. These factors could be important in detecting accurate point estimates and differences, if present. Additionally, populations that provide considerably strong evidence for differential susceptibility among men and women are those that are exposed to higher doses (such as nuclear accident survivors [84, 85] and chronic and acute occupational exposures [86, 87]).

There are several limitations to be acknowledged. Firstly, to assess variability due to exposure characterization we used overlapping buffers ≤ 5 km, ≤ 25 km, and jurisdictional area level comparisons. Ideally, if we had information by nested buffers: ≤ 5 km, > 5—≤ 10 km, > 10—≤ 15 km, and so on, we would have been able to assess if there was a dose–response pattern. However, only two papers [54, 64] included in the meta-analysis provided estimates for 5 km equidistant nested buffer zones, which was insufficient for a meaningful meta-analysis.

We were unable to explore variations in residential proximity to NPPs and thyroid cancer risk by age. Most papers in our analysis provided risk estimates for all ages [39, 54, 63, 65], several did not specify age groups [26, 44, 46, 64, 66] and two papers provided estimates for only adults [33, 47]. Age is an important modifying factor in radiation-induced thyroid cancer as previous analyses of the Chernobyl and Fukushima accident survivors have shown higher sensitivity related to developing thyroid cancer for exposures received at an early age than later in adulthood [88]. A similar pattern was also evident in a pooled analysis of nine cohorts of children exposed to low doses (diagnostically and therapeutically) [89]. The higher susceptibility during childhood may be due to developing cells and faster metabolism [90]. The limited breakdown of risk by age in the identified papers did not allow us to assess variation in risk by age – a key biological risk factor.

Our study has several key strengths including comprehensive literature searching and screening of three indexed databases, reference list search of included papers, and grey literature search. We also conducted subgroup analyses based on key environmental epidemiological principles and current literature to assess heterogeneity in results.

In summary, the results of our meta-analysis suggest a possible modest increase of thyroid cancer incidence for those living near NPPs. Additionally, we also found a stronger effect among studies plausibly prone to biases vs those highly prone to biases and a stronger effect among studies that used smaller buffers vs those that used larger buffers. Our analysis highlights the scarcity of high-quality studies in this research area and the need for future well-designed cohort studies with individual-level data, longer follow-up periods, and accurate exposure characterization.

## Supplementary Information

Supplementary Material 1.

## Data Availability

Data used for the meta-analysis is provided within the manuscript.

## Abbreviations

NPP: Nuclear power plant
TWh: Terawatt-hours
mSv: Millisievert
SIR: Standardized incidence ratio
ASIR: Age-standardized incidence rate
RR: Relative risk
OR: Odds ratio
HR: Hazard risk
CIs: Confidence intervals
PRISMA: Preferred Reporting Items for Systematic Reviews and Meta-analysis
OHAT: Office of Health Assessment and Translation
PROSPERO: The International Prospective Register of Systematic Reviews
PECO: Population, Exposure, Comparator, and Outcomes
NIEHS: National Institute of Environmental Health Sciences
