# Supplementary material for: The risk of thyroid cancer in relation to residential proximity to nuclear power plants: a systematic review and meta-analysis
Source: Environ Health. 2024 Nov 29;23:106. doi: 10.1186/s12940-024-01143-6 (PMC11606113; doi:10.1186/s12940-024-01143-6)
Supplement: Supplementary file 1 — Supplementary Material 1. [file 12940_2024_1143_MOESM1_ESM.docx]

**Supplementary Table 1: Databases and corresponding search terms**

| **Database** | **Search terms** |
| --- | --- |
| SCOPUS | ( TITLE-ABS-KEY ( "nuclear power*"  OR  "nuclear plant*"  OR  ( nuclear  AND  "power plant*" )  OR  "nuclear facilit*"  OR  "nuclear industr*"  OR  "nuclear installation*"  OR  "nuclear site*" )  AND  TITLE-ABS-KEY ( cancer*  OR  neoplasm*  OR  tumor*  OR  tumour*  OR  neoplasia*  OR  carcinoma*  OR  carcinogenesis  OR  malignanc*  OR  adenoma*  OR  sarcoma*  OR  leukemia  OR  melanoma* )  AND NOT  TITLE ( mice*  OR  mouse*  OR  treatment*  OR  accident*  OR  worker* ) )  AND  ( LIMIT-TO ( DOCTYPE ,  "ar" )  OR  LIMIT-TO ( DOCTYPE ,  "sh" ) )  AND  ( LIMIT-TO ( LANGUAGE ,  "English" ) ) |
| WOS | Results for “nuclear power*” OR “nuclear plant*” OR (nuclear AND “power plant*”) OR “nuclear facilit*” OR “nuclear industr*” OR “nuclear installation*” OR “nuclear site*” (Topic) AND cancer* OR neoplasm* OR tumor* OR tumour* OR neoplasia* OR carcinoma* OR carcinogenesis OR malignanc* OR adenoma* OR sarcoma* OR melanoma* OR leukemia* (Topic) NOT mice* OR mouse* OR treatment* OR accident* OR worker* (Title) and Article or Proceeding Paper or Early Access (Document Types) and English (Languages) |
| Pubmed | (("Nuclear Power Plants"[MeSH Terms] OR ("nuclear power*"[Text Word] OR "nuclear plant*"[Text Word] OR ("nuclear"[Text Word] AND "power plant*"[Text Word]) OR ("nuclear facilit*"[All Fields] OR "nuclear industr*"[All Fields] OR "nuclear installation*"[All Fields] OR "nuclear site*"[All Fields]))) AND ("Neoplasms"[MeSH Terms] AND ("cancer*"[Text Word] OR "neoplasm*"[Text Word] OR "tumor*"[Text Word] OR "tumour*"[Text Word] OR "neoplasia*"[Text Word] OR "carcinoma*"[Text Word] OR "carcinogenesis"[Text Word] OR "malignanc*"[Text Word] OR "adenoma*"[Text Word] OR "sarcoma*"[Text Word] OR "melanoma"[Text Word] OR ("leukemia*")))) NOT ("mice*"[Text Word] OR "mouse*"[Text Word] OR "treatment*"[Text Word] OR "accident*"[Text Word] OR "worker*" [Text Word]) NOT (Review[Publication Type]) NOT (Systematic Review[Publication Type]) NOT (Meta-Analysis[Publication Type]) AND (english[Filter]) |

**Supplementary Table 2: OHAT Risk of Bias Rating Tool (for observational studies) – criteria used for scoring**

| **Selection bias** | **Confounding bias** | **Attrition/ exclusion bias** | **Exposure characterization** | **Outcome assessment** | **Selective reporting bias** | **Appropriate Statistical Methods** | **Risk of bias scoring** | |
| --- | --- | --- | --- | --- | --- | --- | --- | --- |
| robust and unbiased methods for selecting subjects or study sites (eg: same source pop) | Relevant individual level variables + area level confounders | minimal and is not associated with the exposure or outcome | Individual dose estimation + residential history (personal dosimeters) | robust data collection processes (eg: from population registry) / well-defined outcome measures | no omission or distortion of findings | Regression (RR/OR/RRR) + good adjustment for confounding / apt model | Definitely low risk of Bias | ++ |
| potential for bias, but the bias is not severe threat to validity | some ecological + some individual variables | some attrition/exclusion but not severe and addressed via analysis | dose estimation (no residential history) | minor concerns but generally reliable and well-structured (very low missing or exclusion form analysis) | minimal instances of selective reporting bias | Regression (RR/OR/RRR) + some adjustment for confounding / SIR/SRR/SMR with ability adjust for confounding | Probably low risk of bias | + |
| significant selection bias (eg: control non-representative) | some area-level ecological variables beyond demographics (eg: SES, area-level smoking) | notable attrition/exclusion bias and affects validity and relability / not addressed in analysis | distance + buffer <20km | inconsistent outcome measures /unreliable data sources | noticeable indications | SIR/SRR/SMR no ability to adjust for confounding | Probably high risk of bias | - |
| severe selection bias (eg: control non-representative) | none/ some area-level ecological variables+ age/sex+region | severe attrition/exclusion bias / not addressed in analysis | areas + vicinity | severe issues in outcome assessment (eg: large missingness or unvalidated data sources) | substantial distortion or suppression of findings | no CI/ Only correlation/description (+ not comparable to other studies) + hypothesis testing | Definitely high risk of bias | -- |
| \| ++ \| Definitely low risk of bias \| \| --- \| --- \| \| + \| Probably low risk of bias \| \| - \| Probably high risk of bias \| \| -- \| Definitely high risk of bias \| | | | | | | | | |

**Supplementary Table 3: Rationale for excluded articles**

| **Lead author** | **Year Published** | **Study design** | **Country/ region** | **Reason** |
| --- | --- | --- | --- | --- |
| Ahn | 2012 | Cohort prospective | Korea | Updated analysis in Kim (2018). Ahn does not provide risk estimates for males |
| Bollaerts | 2014 | Ecological | Belgium | Demoury 2020 includes more years and is adjusted with and better exposure (5km overlapping buffers equidistant buffers vs 0 -20km); same NPPs and same population |
| Bowlt | 1989 | Ecological | UK | Overlapping population with Bunch (2014) |
| COMARE | 2015 | Ecological | UK | Overlapping population and summary estimated in Bunch (2014) |
| Demoury | 2017 | Ecological | Belgium | Demoury 2020 updated analysis for 5km equidistant buffers |
| Kim | 2018 | Cohort + Nested case-control | Korea | Nested case-control excluded due to only one study design (cohort portion included) |
| Kim | 2018 | cohort | Korea | Updated analysis in Kim (2018) |
| Lopez-Abente | 2014 | Ecological | Spain | Cumulative dose and RR no reference population (distance for the cumulative dose not mentioned) - excluded as there is only one such study |
| Mangano | 2009 | Ecological | USA | Cases per 100,000 person and % above the national rate - excluded as there is only one such study |
| Rekacewicz | 1993 | Ecological | France (Chooz) | Overlapping population with Desbiolles 2018 – also Desbiolles 2018 has longer follow-up period and 6 more NPPs |
| Stather | 1984 | Ecological | UK | Risk in terms of probabilities - excluded as there is only one such study |
| Wanigaratne | 2013 | Ecological | Canada | One NPP overlaps with Lane 2013 |

**
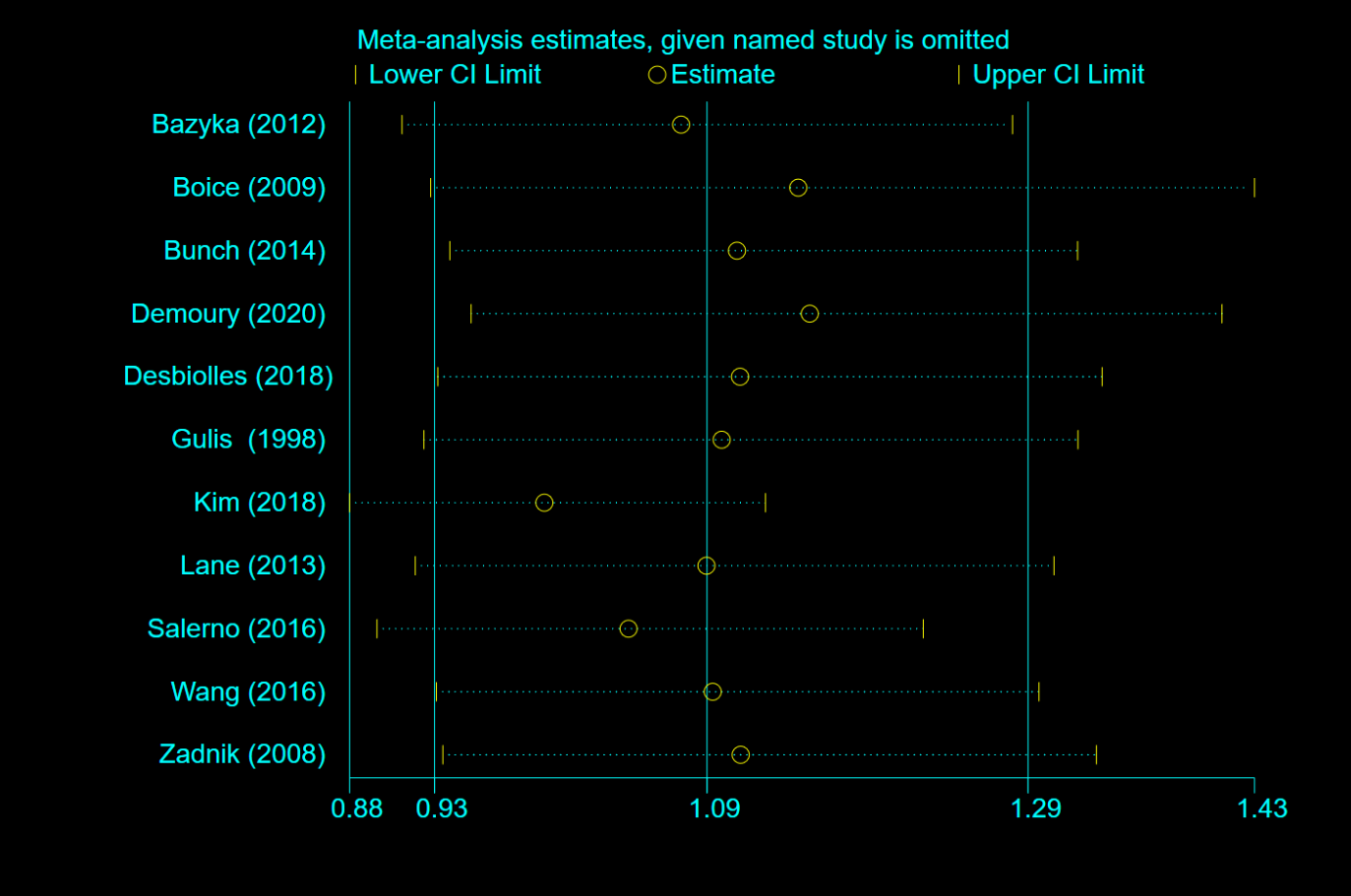
Supplementary Figure 1: Influence analysis for incident thyroid cancer studies among those who live near a nuclear power plant compared to the general population or expected rates using the most commonly reported buffer**
